# 5-(4-Methyl­phen­yl)-2,3-diphenyl-5,6-dihydro­imidazo[1,2-*c*]quinazoline

**DOI:** 10.1107/S1600536810038961

**Published:** 2010-10-09

**Authors:** Jiankang Zhang, Xiaofen Qin, Xingqin Zhou

**Affiliations:** a Key Laboratory of Nuclear Medicine, Ministry of Health, Jiangsu Key Laboratory of Molecular Nuclear Medicine, Jiangsu Institute of Nuclear Medicine, Wuxi, Jiangsu 214063, People’s Republic of China

## Abstract

In the title compound, C_29_H_23_N_3_, the pyrimidine ring adopts an envelope conformation. The dihedral angle between the phenyl rings attached to the pyrimidine-ring double bond is 62.09 (7)°. In the crystal, mol­ecules are linked by N—H⋯N hydrogen bonds, forming extended chains in the *c*-axis direction

## Related literature

For background to quinazolines, see: Blackman *et al.* (1987 ▶); Billimora & Cava (1994 ▶); Helissey *et al.* (1994 ▶); Brana *et al.* (1994 ▶); Riou *et al.* (1991 ▶); Ibrahim *et al.* (1988 ▶); Shi *et al.* (1993 ▶, 2003 ▶); McMurry (1983 ▶). For ttypical C*sp* ^2^—N bond distances, see: Lorente *et al.* (1995 ▶).
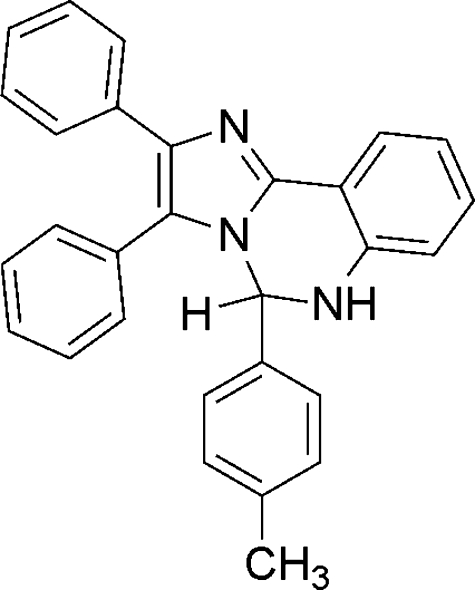


## Experimental

### 

#### Crystal data

C_29_H_23_N_3_
*M* *_r_* = 413.50
Monoclinic, 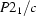

*a* = 16.006 (4) Å
*b* = 11.382 (3) Å
*c* = 11.906 (3) Å
β = 91.810 (4)°
*V* = 2167.8 (10) Å^3^
*Z* = 4
Mo *K*α radiation
μ = 0.08 mm^−1^
*T* = 298 K
0.44 × 0.42 × 0.33 mm

#### Data collection

Bruker SMART CCD area-detector diffractometer
Absorption correction: multi-scan (*SADABS*; Sheldrick, 1996 ▶) *T*_min_ = 0.968, *T*_max_ = 0.976
11276 measured reflections
3819 independent reflections
2071 reflections with *I* > 2σ(*I*)
*R*_int_ = 0.038

#### Refinement

*R*[*F* ^2^ > 2σ(*F* ^2^)] = 0.047
*wR*(*F* ^2^) = 0.146
*S* = 0.95
3819 reflections
289 parameters
H-atom parameters constrained
Δρ_max_ = 0.21 e Å^−3^
Δρ_min_ = −0.25 e Å^−3^

### 

Data collection: *SMART* (Bruker, 1998 ▶); cell refinement: *SAINT* (Bruker, 1999 ▶); data reduction: *SAINT*; program(s) used to solve structure: *SHELXS97* (Sheldrick, 2008 ▶); program(s) used to refine structure: *SHELXL97* (Sheldrick, 2008 ▶); molecular graphics: *SHELXTL* (Sheldrick, 2008 ▶); software used to prepare material for publication: *SHELXTL*.

## Supplementary Material

Crystal structure: contains datablocks global, I. DOI: 10.1107/S1600536810038961/vm2044sup1.cif

Structure factors: contains datablocks I. DOI: 10.1107/S1600536810038961/vm2044Isup2.hkl

Additional supplementary materials:  crystallographic information; 3D view; checkCIF report

## Figures and Tables

**Table 1:** Hydrogen-bond geometry (Å, °)

*D*—H⋯*A*	*D*—H	H⋯*A*	*D*⋯*A*	*D*—H⋯*A*
N1—H1*A*⋯N3^i^	0.86	2.46	3.058 (3)	127

Symmetry code: (i) 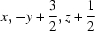.

**Fig. 1.**
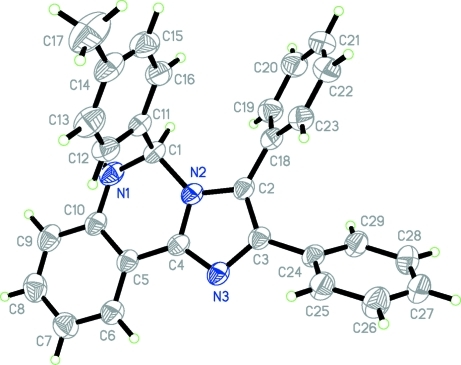
The molecular structure of (I), showing 40% probability displacement ellipsoids and the atom-numbering scheme.

**Fig. 2.**
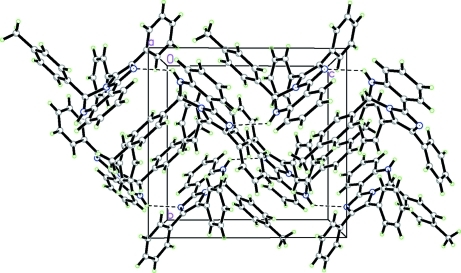
The crystal packing of (I).

## supplementary crystallographic information

### Comment

Quinazolines are an important class of compounds found in many natural products (*e.g.* hinckdentin A, Blackman *et al.*, 1987; Billimora & Cava, 1994), and employed as potent agents (Helissey *et al.*, 1994; Brana *et al.*, 1994; Riou *et al.*, 1991; Ibrahim *et al.*, 1988). Low-valent titanium reagents have an exceedingly high ability to promote reductive coupling of carbonyl compounds and are attracting increasing interest in organic synthesis (McMurry, 1983; Shi *et al.*, 1993; 2003).

We report here the crystal structure of the title compound, (I), which was synthesized by the reaction of 4,5-diphenyl-2-(2-nitrophenyl)imidazole with 4-methylbenzaldehyde oxime, induced by low-valent titanium reagent (TiCl_4_/Sm) using THF as solvent at refluxing temperature.

In (I), atoms N1, C1, N2, C4, C5 and C10 form a fused pyrimidine ring, with interatomic distances of 1.445 (3)Å for N1—C1 and 1.475 (3)Å for N2—C1, which indicate that these C—N bonds are single. The pyrimidine ring adopts an envelope conformation; atoms N1, N2, C4, C5 and C10 are coplanar, while atom C1 deviates from this plane by -0.383 (3) Å. The dihedral angle between the C18—C23 and C24—C29 phenyl rings is 62.09 (7)°. In addition, because of the existence of a conjugated system, the N1—C10 [1.384 (3) Å], N2—C2 [1.382 (3) Å] and N2—C4 [1.362 (3) Å] distances are significantly shorter than the typical C*sp*^2^—N bond distnec (1.426 Å; Lorente *et al.*, 1995). The molecules are linked by N—H···N hydrogen bonds to form extended chains in the c direction (Table 1, Fig. 2).

### Experimental

The title compound, (I), was prepared by the reaction of 4,5-diphenyl-2-(2-nitrophenyl)imidazole with 4-methylbenzaldehyde oxime, induced by low-valent titanium reagent (TiCl_4_/Sm). The single crystals of (I) suitable for X-ray diffraction were obtained by slow evaporation of a ethanol solution. ^1^H NMR (DMSO-d_6_, δ): 2.18 (3*H*, s, CH_3_), 6.32 (1*H*, d, J = 2.4 Hz, CH), 6.72 (2*H*, d, J = 8.4 Hz, ArH), 6.77–6.82 (2*H*, m, ArH), 7.01 (2*H*, d, J = 8.0 Hz, ArH), 7.09–7.26 (6*H*, m, ArH), 7.33 (1*H*, d, J = 2.4 Hz, ArH), 7.38–7.44 (3*H*, m, ArH), 7.51 (2*H*, d, J = 8.8 Hz, ArH), 7.84 (1*H*, d, J = 7.6 Hz, NH).

### Refinement

H atoms were positioned geometrically and treated as riding on their parent atoms, with C—H distances in the range 0.93–0.98Å and N—H distance of 0.86 Å; the *U*_iso_(H) values were set equal to 1.2–1.5*U*_eq_(C).

### Crystal data

**Table:**

C_29_H_23_N_3_	*F*(000) = 872
*M**_r_* = 413.50	*D*_x_ = 1.267 Mg m^−^^3^
Monoclinic, *P*2_1_/*c*	Mo *K*α radiation, λ = 0.71073 Å
Hall symbol: -P 2ybc	Cell parameters from 2039 reflections
*a* = 16.006 (4) Å	θ = 2.6–23.2°
*b* = 11.382 (3) Å	µ = 0.08 mm^−^^1^
*c* = 11.906 (3) Å	*T* = 298 K
β = 91.810 (4)°	Block, yellow
*V* = 2167.8 (10) Å^3^	0.44 × 0.42 × 0.33 mm
*Z* = 4	

### Data collection

**Table:**

Bruker SMART CCD area-detector diffractometer	3819 independent reflections
Radiation source: fine-focus sealed tube	2071 reflections with *I* > 2σ(*I*)
graphite	*R*_int_ = 0.038
φ and ω scans	θ_max_ = 25.0°, θ_min_ = 2.2°
Absorption correction: multi-scan (*SADABS*; Sheldrick, 1996)	*h* = −18→19
*T*_min_ = 0.968, *T*_max_ = 0.976	*k* = −10→13
11276 measured reflections	*l* = −14→14

### Refinement

**Table:**

Refinement on *F*^2^	Primary atom site location: structure-invariant direct methods
Least-squares matrix: full	Secondary atom site location: difference Fourier map
*R*[*F*^2^ > 2σ(*F*^2^)] = 0.047	Hydrogen site location: inferred from neighbouring sites
*wR*(*F*^2^) = 0.146	H-atom parameters constrained
*S* = 0.95	*w* = 1/[σ^2^(*F*_o_^2^) + (0.0596*P*)^2^ + 0.8501*P*] where *P* = (*F*_o_^2^ + 2*F*_c_^2^)/3
3819 reflections	(Δ/σ)_max_ < 0.001
289 parameters	Δρ_max_ = 0.21 e Å^−^^3^
0 restraints	Δρ_min_ = −0.24 e Å^−^^3^

### Special details

**Table:**

Geometry. All e.s.d.'s (except the e.s.d. in the dihedral angle between two l.s. planes) are estimated using the full covariance matrix. The cell e.s.d.'s are taken into account individually in the estimation of e.s.d.'s in distances, angles and torsion angles; correlations between e.s.d.'s in cell parameters are only used when they are defined by crystal symmetry. An approximate (isotropic) treatment of cell e.s.d.'s is used for estimating e.s.d.'s involving l.s. planes.
Refinement. Refinement of *F*^2^ against ALL reflections. The weighted *R*-factor *wR* and goodness of fit *S* are based on *F*^2^, conventional *R*-factors *R* are based on *F*, with *F* set to zero for negative *F*^2^. The threshold expression of *F*^2^ > σ(*F*^2^) is used only for calculating *R*-factors(gt) *etc*. and is not relevant to the choice of reflections for refinement. *R*-factors based on *F*^2^ are statistically about twice as large as those based on *F*, and *R*- factors based on ALL data will be even larger.

### Fractional atomic coordinates and isotropic or equivalent isotropic displacement parameters (Å^2^)

**Table:**

	*x*	*y*	*z*	*U*_iso_*/*U*_eq_	
N1	0.14553 (14)	0.83172 (18)	0.85068 (18)	0.0517 (6)	
H1A	0.1495	0.8943	0.8910	0.062*	
N2	0.21849 (13)	0.69225 (17)	0.74512 (16)	0.0418 (5)	
N3	0.16675 (13)	0.59511 (17)	0.59709 (16)	0.0429 (5)	
C1	0.21383 (16)	0.7487 (2)	0.8563 (2)	0.0442 (6)	
H1	0.2657	0.7927	0.8704	0.053*	
C2	0.28310 (15)	0.6234 (2)	0.7078 (2)	0.0421 (6)	
C3	0.24973 (16)	0.5649 (2)	0.6166 (2)	0.0423 (6)	
C4	0.15000 (16)	0.6717 (2)	0.67723 (19)	0.0405 (6)	
C5	0.07381 (16)	0.7354 (2)	0.6952 (2)	0.0422 (6)	
C6	0.00388 (17)	0.7246 (2)	0.6248 (2)	0.0513 (7)	
H6	0.0039	0.6710	0.5658	0.062*	
C7	−0.06578 (18)	0.7928 (3)	0.6415 (3)	0.0618 (8)	
H7	−0.1127	0.7855	0.5939	0.074*	
C8	−0.06544 (19)	0.8721 (3)	0.7295 (3)	0.0640 (8)	
H8	−0.1124	0.9181	0.7407	0.077*	
C9	0.00313 (19)	0.8838 (2)	0.8003 (2)	0.0576 (8)	
H9	0.0024	0.9371	0.8594	0.069*	
C10	0.07411 (17)	0.8157 (2)	0.7839 (2)	0.0455 (7)	
C11	0.20700 (16)	0.6564 (2)	0.9477 (2)	0.0435 (6)	
C12	0.13376 (18)	0.5960 (2)	0.9621 (2)	0.0557 (7)	
H12	0.0878	0.6108	0.9144	0.067*	
C13	0.1277 (2)	0.5138 (2)	1.0465 (2)	0.0653 (8)	
H13	0.0775	0.4742	1.0552	0.078*	
C14	0.1946 (2)	0.4892 (2)	1.1179 (2)	0.0602 (8)	
C15	0.2675 (2)	0.5504 (3)	1.1031 (2)	0.0650 (8)	
H15	0.3134	0.5355	1.1508	0.078*	
C16	0.27413 (18)	0.6330 (2)	1.0196 (2)	0.0557 (7)	
H16	0.3242	0.6732	1.0116	0.067*	
C17	0.1870 (3)	0.4005 (3)	1.2109 (3)	0.0974 (13)	
H17A	0.1699	0.4394	1.2779	0.146*	
H17B	0.2401	0.3631	1.2249	0.146*	
H17C	0.1461	0.3424	1.1890	0.146*	
C18	0.36672 (16)	0.6215 (2)	0.7620 (2)	0.0451 (6)	
C19	0.41102 (18)	0.7239 (3)	0.7841 (2)	0.0558 (7)	
H19	0.3885	0.7957	0.7613	0.067*	
C20	0.48788 (19)	0.7208 (3)	0.8393 (3)	0.0666 (9)	
H20	0.5163	0.7904	0.8552	0.080*	
C21	0.5223 (2)	0.6158 (3)	0.8708 (3)	0.0735 (10)	
H21	0.5742	0.6137	0.9083	0.088*	
C22	0.48048 (19)	0.5135 (3)	0.8470 (3)	0.0669 (9)	
H22	0.5046	0.4419	0.8673	0.080*	
C23	0.40312 (17)	0.5155 (3)	0.7934 (2)	0.0559 (7)	
H23	0.3751	0.4454	0.7781	0.067*	
C24	0.29181 (16)	0.4794 (2)	0.5439 (2)	0.0442 (6)	
C25	0.25755 (18)	0.3695 (2)	0.5252 (2)	0.0576 (8)	
H25	0.2068	0.3504	0.5563	0.069*	
C26	0.2981 (2)	0.2877 (3)	0.4605 (3)	0.0681 (9)	
H26	0.2755	0.2131	0.4501	0.082*	
C27	0.3716 (2)	0.3166 (3)	0.4118 (3)	0.0672 (9)	
H27	0.3985	0.2619	0.3677	0.081*	
C28	0.40545 (19)	0.4259 (3)	0.4280 (2)	0.0649 (8)	
H28	0.4551	0.4457	0.3942	0.078*	
C29	0.36592 (17)	0.5066 (2)	0.4945 (2)	0.0551 (7)	
H29	0.3897	0.5804	0.5061	0.066*	

### Atomic displacement parameters (Å^2^)

**Table:**

	*U*^11^	*U*^22^	*U*^33^	*U*^12^	*U*^13^	*U*^23^
N1	0.0703 (16)	0.0421 (13)	0.0425 (13)	0.0085 (11)	0.0006 (12)	−0.0063 (10)
N2	0.0491 (13)	0.0440 (12)	0.0324 (11)	0.0006 (10)	0.0024 (10)	0.0010 (9)
N3	0.0507 (14)	0.0441 (12)	0.0340 (12)	0.0021 (10)	0.0050 (10)	0.0018 (10)
C1	0.0535 (16)	0.0445 (15)	0.0346 (14)	−0.0002 (12)	0.0033 (12)	−0.0062 (11)
C2	0.0477 (15)	0.0454 (15)	0.0334 (14)	0.0028 (12)	0.0050 (12)	0.0000 (12)
C3	0.0496 (16)	0.0445 (15)	0.0329 (14)	0.0021 (12)	0.0036 (12)	0.0033 (11)
C4	0.0503 (16)	0.0407 (14)	0.0305 (13)	−0.0015 (12)	0.0033 (12)	0.0042 (11)
C5	0.0512 (16)	0.0418 (15)	0.0339 (14)	0.0035 (12)	0.0053 (12)	0.0050 (11)
C6	0.0566 (18)	0.0525 (17)	0.0451 (16)	0.0021 (14)	0.0042 (14)	0.0006 (13)
C7	0.0556 (19)	0.068 (2)	0.062 (2)	0.0059 (15)	−0.0002 (15)	0.0038 (16)
C8	0.063 (2)	0.064 (2)	0.066 (2)	0.0167 (16)	0.0109 (17)	0.0087 (17)
C9	0.075 (2)	0.0488 (17)	0.0493 (17)	0.0156 (15)	0.0106 (16)	0.0018 (13)
C10	0.0578 (17)	0.0447 (16)	0.0344 (14)	0.0039 (13)	0.0066 (13)	0.0068 (12)
C11	0.0543 (17)	0.0443 (15)	0.0320 (14)	0.0011 (13)	0.0039 (12)	−0.0035 (11)
C12	0.0635 (19)	0.0603 (19)	0.0433 (16)	−0.0044 (15)	0.0001 (14)	0.0024 (14)
C13	0.086 (2)	0.0592 (19)	0.0513 (18)	−0.0121 (16)	0.0100 (17)	0.0038 (15)
C14	0.094 (2)	0.0461 (17)	0.0404 (17)	0.0115 (17)	0.0076 (17)	0.0023 (13)
C15	0.081 (2)	0.067 (2)	0.0462 (18)	0.0185 (18)	−0.0051 (16)	0.0047 (15)
C16	0.0615 (19)	0.0587 (18)	0.0466 (17)	0.0031 (14)	−0.0012 (14)	−0.0018 (14)
C17	0.170 (4)	0.064 (2)	0.060 (2)	0.014 (2)	0.019 (2)	0.0197 (17)
C18	0.0500 (16)	0.0529 (17)	0.0327 (14)	0.0012 (13)	0.0044 (12)	0.0007 (12)
C19	0.0572 (18)	0.0603 (19)	0.0500 (17)	−0.0040 (15)	0.0028 (14)	0.0028 (14)
C20	0.057 (2)	0.081 (2)	0.062 (2)	−0.0118 (17)	0.0015 (16)	−0.0113 (17)
C21	0.0548 (19)	0.104 (3)	0.061 (2)	0.008 (2)	−0.0094 (16)	−0.0104 (19)
C22	0.064 (2)	0.075 (2)	0.061 (2)	0.0153 (17)	−0.0085 (16)	−0.0006 (17)
C23	0.0582 (19)	0.0602 (19)	0.0491 (17)	0.0064 (15)	−0.0012 (14)	−0.0014 (14)
C24	0.0511 (16)	0.0485 (16)	0.0329 (14)	0.0051 (13)	0.0009 (12)	0.0003 (12)
C25	0.0598 (18)	0.0563 (18)	0.0571 (18)	−0.0021 (14)	0.0085 (15)	−0.0087 (14)
C26	0.078 (2)	0.0559 (19)	0.071 (2)	0.0020 (16)	0.0070 (19)	−0.0143 (16)
C27	0.070 (2)	0.072 (2)	0.059 (2)	0.0176 (18)	0.0056 (17)	−0.0183 (16)
C28	0.0555 (18)	0.082 (2)	0.0580 (19)	0.0029 (17)	0.0165 (15)	−0.0093 (17)
C29	0.0583 (19)	0.0591 (18)	0.0484 (17)	−0.0034 (14)	0.0104 (15)	−0.0071 (14)

### Geometric parameters (Å, °)

**Table:**

N1—C10	1.384 (3)	C14—C15	1.374 (4)
N1—C1	1.445 (3)	C14—C17	1.506 (4)
N1—H1A	0.8600	C15—C16	1.375 (4)
N2—C4	1.362 (3)	C15—H15	0.9300
N2—C2	1.382 (3)	C16—H16	0.9300
N2—C1	1.475 (3)	C17—H17A	0.9600
N3—C4	1.326 (3)	C17—H17B	0.9600
N3—C3	1.384 (3)	C17—H17C	0.9600
C1—C11	1.519 (3)	C18—C23	1.387 (3)
C1—H1	0.9800	C18—C19	1.385 (4)
C2—C3	1.368 (3)	C19—C20	1.377 (4)
C2—C18	1.468 (3)	C19—H19	0.9300
C3—C24	1.478 (3)	C20—C21	1.365 (4)
C4—C5	1.440 (3)	C20—H20	0.9300
C5—C6	1.383 (3)	C21—C22	1.368 (4)
C5—C10	1.396 (3)	C21—H21	0.9300
C6—C7	1.378 (4)	C22—C23	1.375 (4)
C6—H6	0.9300	C22—H22	0.9300
C7—C8	1.382 (4)	C23—H23	0.9300
C7—H7	0.9300	C24—C29	1.376 (3)
C8—C9	1.369 (4)	C24—C25	1.381 (4)
C8—H8	0.9300	C25—C26	1.384 (4)
C9—C10	1.394 (4)	C25—H25	0.9300
C9—H9	0.9300	C26—C27	1.366 (4)
C11—C12	1.374 (4)	C26—H26	0.9300
C11—C16	1.379 (3)	C27—C28	1.368 (4)
C12—C13	1.379 (4)	C27—H27	0.9300
C12—H12	0.9300	C28—C29	1.380 (4)
C13—C14	1.374 (4)	C28—H28	0.9300
C13—H13	0.9300	C29—H29	0.9300
			
C10—N1—C1	123.4 (2)	C15—C14—C17	121.5 (3)
C10—N1—H1A	118.3	C13—C14—C17	120.8 (3)
C1—N1—H1A	118.3	C14—C15—C16	121.6 (3)
C4—N2—C2	108.0 (2)	C14—C15—H15	119.2
C4—N2—C1	123.0 (2)	C16—C15—H15	119.2
C2—N2—C1	126.5 (2)	C15—C16—C11	120.3 (3)
C4—N3—C3	104.9 (2)	C15—C16—H16	119.8
N1—C1—N2	107.7 (2)	C11—C16—H16	119.8
N1—C1—C11	114.4 (2)	C14—C17—H17A	109.5
N2—C1—C11	110.40 (19)	C14—C17—H17B	109.5
N1—C1—H1	108.1	H17A—C17—H17B	109.5
N2—C1—H1	108.1	C14—C17—H17C	109.5
C11—C1—H1	108.1	H17A—C17—H17C	109.5
C3—C2—N2	104.7 (2)	H17B—C17—H17C	109.5
C3—C2—C18	132.0 (2)	C23—C18—C19	118.2 (3)
N2—C2—C18	123.2 (2)	C23—C18—C2	120.0 (2)
C2—C3—N3	111.1 (2)	C19—C18—C2	121.7 (2)
C2—C3—C24	127.7 (2)	C20—C19—C18	120.9 (3)
N3—C3—C24	121.2 (2)	C20—C19—H19	119.5
N3—C4—N2	111.3 (2)	C18—C19—H19	119.5
N3—C4—C5	129.0 (2)	C21—C20—C19	120.0 (3)
N2—C4—C5	119.7 (2)	C21—C20—H20	120.0
C6—C5—C10	120.0 (2)	C19—C20—H20	120.0
C6—C5—C4	122.5 (2)	C22—C21—C20	119.9 (3)
C10—C5—C4	117.4 (2)	C22—C21—H21	120.1
C7—C6—C5	120.4 (3)	C20—C21—H21	120.1
C7—C6—H6	119.8	C21—C22—C23	120.7 (3)
C5—C6—H6	119.8	C21—C22—H22	119.7
C6—C7—C8	119.5 (3)	C23—C22—H22	119.7
C6—C7—H7	120.2	C22—C23—C18	120.2 (3)
C8—C7—H7	120.2	C22—C23—H23	119.9
C9—C8—C7	120.9 (3)	C18—C23—H23	119.9
C9—C8—H8	119.5	C29—C24—C25	118.6 (2)
C7—C8—H8	119.5	C29—C24—C3	121.1 (2)
C8—C9—C10	120.0 (3)	C25—C24—C3	120.3 (2)
C8—C9—H9	120.0	C24—C25—C26	120.6 (3)
C10—C9—H9	120.0	C24—C25—H25	119.7
N1—C10—C5	120.2 (2)	C26—C25—H25	119.7
N1—C10—C9	120.6 (2)	C27—C26—C25	119.9 (3)
C5—C10—C9	119.1 (3)	C27—C26—H26	120.0
C12—C11—C16	118.5 (2)	C25—C26—H26	120.0
C12—C11—C1	121.1 (2)	C26—C27—C28	120.1 (3)
C16—C11—C1	120.4 (2)	C26—C27—H27	119.9
C11—C12—C13	120.6 (3)	C28—C27—H27	119.9
C11—C12—H12	119.7	C27—C28—C29	120.0 (3)
C13—C12—H12	119.7	C27—C28—H28	120.0
C14—C13—C12	121.2 (3)	C29—C28—H28	120.0
C14—C13—H13	119.4	C24—C29—C28	120.8 (3)
C12—C13—H13	119.4	C24—C29—H29	119.6
C15—C14—C13	117.7 (3)	C28—C29—H29	119.6
			
C10—N1—C1—N2	31.9 (3)	N1—C1—C11—C12	47.4 (3)
C10—N1—C1—C11	−91.3 (3)	N2—C1—C11—C12	−74.2 (3)
C4—N2—C1—N1	−32.5 (3)	N1—C1—C11—C16	−131.4 (3)
C2—N2—C1—N1	167.7 (2)	N2—C1—C11—C16	106.9 (3)
C4—N2—C1—C11	93.1 (3)	C16—C11—C12—C13	−0.1 (4)
C2—N2—C1—C11	−66.8 (3)	C1—C11—C12—C13	−179.0 (2)
C4—N2—C2—C3	0.7 (3)	C11—C12—C13—C14	−0.5 (4)
C1—N2—C2—C3	163.1 (2)	C12—C13—C14—C15	0.7 (4)
C4—N2—C2—C18	−179.2 (2)	C12—C13—C14—C17	179.1 (3)
C1—N2—C2—C18	−16.9 (4)	C13—C14—C15—C16	−0.4 (4)
N2—C2—C3—N3	−0.3 (3)	C17—C14—C15—C16	−178.8 (3)
C18—C2—C3—N3	179.7 (2)	C14—C15—C16—C11	−0.2 (4)
N2—C2—C3—C24	179.8 (2)	C12—C11—C16—C15	0.4 (4)
C18—C2—C3—C24	−0.3 (5)	C1—C11—C16—C15	179.3 (2)
C4—N3—C3—C2	−0.3 (3)	C3—C2—C18—C23	−52.0 (4)
C4—N3—C3—C24	179.7 (2)	N2—C2—C18—C23	127.9 (3)
C3—N3—C4—N2	0.8 (3)	C3—C2—C18—C19	128.5 (3)
C3—N3—C4—C5	177.5 (2)	N2—C2—C18—C19	−51.6 (4)
C2—N2—C4—N3	−1.0 (3)	C23—C18—C19—C20	−2.3 (4)
C1—N2—C4—N3	−164.1 (2)	C2—C18—C19—C20	177.2 (2)
C2—N2—C4—C5	−178.0 (2)	C18—C19—C20—C21	1.5 (4)
C1—N2—C4—C5	18.9 (3)	C19—C20—C21—C22	0.2 (5)
N3—C4—C5—C6	−0.9 (4)	C20—C21—C22—C23	−1.2 (5)
N2—C4—C5—C6	175.5 (2)	C21—C22—C23—C18	0.4 (5)
N3—C4—C5—C10	−177.0 (2)	C19—C18—C23—C22	1.3 (4)
N2—C4—C5—C10	−0.5 (3)	C2—C18—C23—C22	−178.2 (3)
C10—C5—C6—C7	0.0 (4)	C2—C3—C24—C29	−53.1 (4)
C4—C5—C6—C7	−176.0 (2)	N3—C3—C24—C29	127.0 (3)
C5—C6—C7—C8	−0.1 (4)	C2—C3—C24—C25	126.2 (3)
C6—C7—C8—C9	−0.1 (4)	N3—C3—C24—C25	−53.7 (3)
C7—C8—C9—C10	0.4 (4)	C29—C24—C25—C26	1.7 (4)
C1—N1—C10—C5	−17.9 (4)	C3—C24—C25—C26	−177.7 (3)
C1—N1—C10—C9	165.5 (2)	C24—C25—C26—C27	−1.9 (4)
C6—C5—C10—N1	−176.3 (2)	C25—C26—C27—C28	0.7 (5)
C4—C5—C10—N1	−0.2 (3)	C26—C27—C28—C29	0.7 (5)
C6—C5—C10—C9	0.4 (4)	C25—C24—C29—C28	−0.2 (4)
C4—C5—C10—C9	176.5 (2)	C3—C24—C29—C28	179.1 (2)
C8—C9—C10—N1	176.1 (2)	C27—C28—C29—C24	−0.9 (4)
C8—C9—C10—C5	−0.5 (4)		

### Hydrogen-bond geometry (Å, °)

**Table:**

*D*—H···*A*	*D*—H	H···*A*	*D*···*A*	*D*—H···*A*
N1—H1A···N3^i^	0.86	2.46	3.058 (3)	127

Symmetry codes: (i) *x*, −*y*+3/2, *z*+1/2.
